# Monitoring Group 2 Innate Lymphoid Cell Biology in Models of Lung Inflammation

**DOI:** 10.21769/BioProtoc.4717

**Published:** 2023-07-20

**Authors:** Jana H. Badrani, Allyssa N. Strohm, Yung-An Haung, Taylor A. Doherty

**Affiliations:** 1 Divison of Rheumatology, Allergy and Immunology, Department of Medicine, University of California San Diego, La Jolla, CA, USA; 2 Veterans Affairs San Diego Health Care System, La Jolla, CA, USA

**Keywords:** Innate lymphoid cells, Lung inflammation, *Alternaria alternata*, Flow cytometry, Intracellular staining

## Abstract

Innate lymphoid cells (ILCs) are a rare cell population subdivided into ILC1s, ILC2s, and ILC3s, based on transcription factor expression and cytokine production. In models of lung inflammation, the release of alarmins from the epithelium activates ILC2s and promotes the production of Th2-cytokines and the proliferation and migration of ILC2s within the lung. ILC2s are the innate counterpart to CD4^+^ Th2s and, as such, express Gata-3 and produce IL-4, IL-5, and IL-13. Due to the low number of ILCs and the lack of specific surface markers, flow cytometry is the most reliable technique for the identification and characterization of ILCs. In this protocol, multicolor flow cytometry is utilized to identify Lineage- Thy1.2+ ILCs. Intracellular cytokine staining further identifies ILC2s within the lung. This protocol presents a reliable method for promoting ILC2-mediated lung inflammation and for monitoring ILC2 biology.

Key features

In this protocol, ILC2s are expanded via intranasal challenges with*Alternaria alternata*, a fungal allergen, or recombinant IL-33.

Bronchoalveolar lavage (BAL) and lung are collected and processed into single-cell suspension for multicolor flow cytometric analysis, including intracellular staining of transcription factors and cytokines.

During lung inflammation, the percentage of ILC2s and eosinophils increases. ILC2s express greater levels of*Gata-3*and*Ki-67*and produce greater amounts of IL-5 and IL-13.

Graphical overview

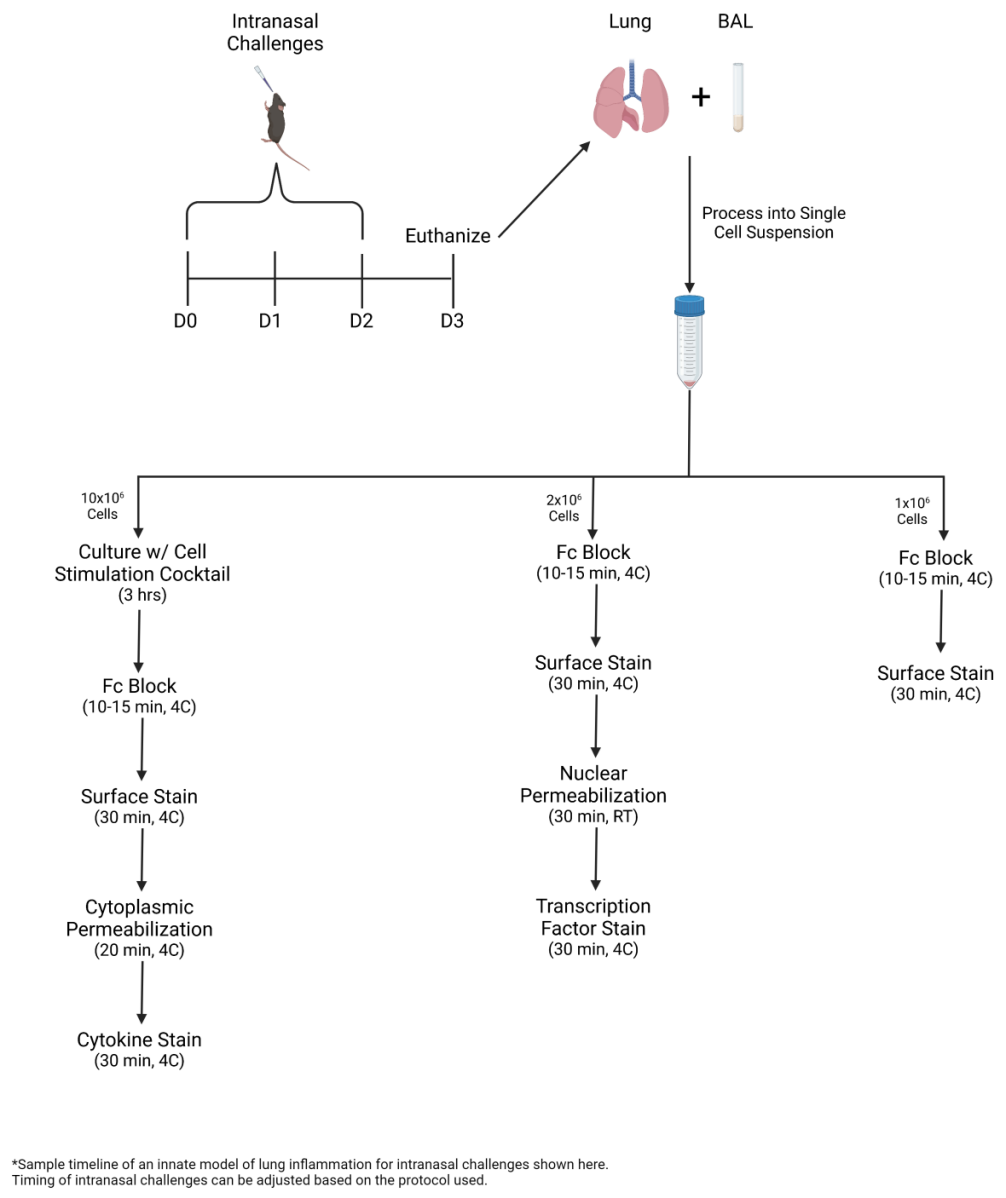

## Background

Innate lymphoid cells (ILCs) are a heterogeneous population of lymphocytes that lack common lineage markers. ILCs are differentiated by their transcription factor expression profiles and cytokine production capabilities (Wirtz et al., 2021). ILCs are categorized into three major subpopulations, ILC1s, ILC2s, and ILC3s, which are the innate counterparts to CD4^+^Th1, Th2, and Th17 cells (Artis and Spits, 2015). ILCs and their T-cell counterparts share similar functions, transcription factor profiles, and cytokine production. ILC1s and Th1s express T-bet and produce IFNγ in response to intracellular pathogens. ILC2s and Th2s express Gata-3 and produce IL-4, IL-5, and IL-13 in response to extracellular pathogens and allergens. ILC3s and Th17s express RORγt and produce IL-17a in response to extracellular pathogens (Drake et al., 2014;Vivier et al., 2018).

In the lung, ILC2s are the most common ILC subpopulation (Gasteiger et al., 2015). Lung-resident ILC2s can be identified by their expression of Thy-1, CD127 (IL-7 receptor), CD25 (IL-2 receptor), and T1ST2 (IL-33 receptor) (Monticelli et al., 2011). Lung inflammation is associated with the epithelial release of alarmins, including IL-33, IL-25, and thymic stromal lymphopoietin (Rossi et al., 2022). ILC2s respond to alarmins and mediate inflammation within the lungs; in response to environmental allergens, ILC2s produce their effector cytokines and expand locally (Lai et al., 2016). ILC2-derived IL-4 is important for the differentiation of naïve T cells into Th2 cells and the transition to adaptive immunity (Pelly et al., 2016). IL-5 plays a role in maintaining and promoting eosinophils (Ikutani et al., 2017), while IL-13 promotes mucus secretion (Varela et al., 2022). With inflammation, lung ILC2s are more motile and accumulate in peribronchial and perivascular spaces (Puttur et al., 2019).

In addition to being a rare cell population, ILCs lack specific and unique surface markers, which presents a challenge in identifying ILCs in tissues. Flow cytometry is the most established technique for identifying and characterizing ILCs, including the different subpopulations. Multicolor flow cytometry allows for the identification of multiple populations based on multiple parameters. For ILC populations, a combination of surface markers, transcription factors, and cytokines serves as the basis for identifying these populations. Here, murine ILCs are identified as Lineage- Thy1.2^+^lymphocytes. IL-5 and IL-13 production is used to further identify pulmonary ILC2s. In this protocol, ILC2s are expanded via intranasal challenges with*Alternaria alternata*, a fungal allergen, or recombinant IL-33. Bronchoalveolar lavage and lung are collected and processed into single-cell suspension for multicolor flow cytometric analysis, including intracellular staining of transcription factors and cytokines.

## Materials and reagents

1. 21 G needle (BD, catalog number: 305167)
2. 18 G needle (BD, catalog number: 305196)
3. 1.5 mL Eppendorf tubes (Genesee Scientific, catalog number: 24-282S)
4. Flat-bottom 96-well plate (Genesee Scientific, catalog number: 25-109)
5. 70 μm filter (Corning, catalog number: 431751)
6. 15 mL conical tubes (FroggaBio, catalog number: TB15-25)
7. 50 mL conical tubes (Falcon, catalog number: 352098)
8. Suture (Look, catalog number: SP105)
9. gentleMACS c-tube (Miltenyi Biotec, catalog number: 130-093-237)
10. Polystyrene FACS tubes (Fisher Scientific, catalog number: 14-959-5)
11. C57BL/6 mice (6–8 weeks old; female)
12. Miltenyi Lung Digest kit (Miltenyi Biotec, catalog number: 130-095-927)
13. Mouse recombinant IL-33 (Thermo Fisher, catalog number: 14-8332-80)
14. *Alternaria alternata*extract (Fisher Scientific, catalog number: NC1620293)
15. 1× PBS (Gibco, catalog number: 10010-023)
16. RPMI (Gibco, catalog number: 21870-076)
17. Bovine serum albumin (BSA) (Sigma-Aldrich, catalog number: A7906-100G)
18. Fetal bovine serum (FBS) (Corning, catalog number: 35-015-CV)
19. Sodium azide (Fisher Scientific, catalog number: BP922I-500)
20. Penicillin/streptavidin (Gibco, catalog number: 15140122)
21. L-Glutamine (Gibco, catalog number: 25030-081)
22. β-Mercaptoethanol (Gibco, catalog number: 21985-023)
23. Cell stimulation cocktail plus protein transport inhibitor (Thermo Fisher Scientific, catalog number: 00-4975-93)
24. PE anti-mouse IL-13 (Thermo Fisher, catalog number: 12-7133-82; clone: eBio13A, dilution: 1:20)
25. PE anti-mouse/human IL-5 (BioLegend, catalog number: 504304; clone: TRFK5, dilution: 1:20)
26. PE Ki-67 monoclonal antibody (Thermo Fisher, catalog number: 12-5698-82; clone: SolA15, dilution: 1:50)
27. APC anti-mouse Ly6G-Ly6C (GR1) (BioLegend, catalog number: 108412; clone: RB6-8C5, dilution: 1:50)
28. PE anti-mouse SiglecF (BD Biosciences, catalog number: 552126; clone: E502440, dilution: 1:50)
29. PerCP anti-mouse CD45.2 (BioLegend, catalog number: 109828; clone: 104, dilution: 1:50)
30. FITC anti-mouse TCR γ/δ (BioLegend, catalog number: 118106; clone: GL3, dilution: 1:200)
31. FITC anti-mouse TCR β Chain (BioLegend, catalog number: 109206; clone: H57-597, dilution: 1:200)
32. FITC anti-mouse FceR1α (BioLegend, catalog number: 134306; clone: MAR-1, dilution: 1:200)
33. FITC anti-mouse CD5 (BioLegend, catalog number: 100606; clone: 53-7.3, dilution: 1:200)
34. FITC anti-mouse NK-1.1 (BioLegend, catalog number: 108706; clone: PK136, dilution: 1:200)
35. FITC anti-mouse CD11c (BioLegend, catalog number: 117306; clone: N418, dilution: 1:200)
36. FITC anti-mouse Lineage (BioLegend, catalog number: 133302; clone: 145-2C11; RB6-8C5; RA3-6B2; Ter-119; M1/70, dilution: 1:40)
37. Purified anti-mouse CD16/32 (Fc Block) (BioLegend, catalog number:101302; clone: 93, dilution: 1:50)
38. BD Cytofix/Cytoperm Fixation/Permeabilization kit (Fisher Scientific, catalog number: BDB554714)
39. FoxP3/Transcription Factor Staining Buffer Set (FoxP3 Perm kit and buffer) (ThermoFisher, catalog number: 00-5523-00)
40. 32% paraformaldehyde (PFA), EM grade (Electron Microscopy Sciences, catalog number: 15714) (diluted to 4% with DI water)
41. FACS buffer (see Recipes)
42. T-cell media (see Recipes)

## Equipment

1. Single-channel pipettes (Rainin: P10, P200, P1000)
2. Multi-channel pipette (Thermo Scientific)
3. Catheter tubing (BD, catalog number: 427411)
4. Fine forceps (Fine Surgical Tools, catalog number: 11412-11)
5. Curved forceps (Fine Surgical Tools, catalog number: 11054-10)
6. Surgical scissors (Fine Surgical Tools, catalog number: 14060-10)
7. Centrifuge (Beckman Coulter, model: Allegra X-15R, swinging bucket rotor)
8. gentleMACS dissociator, octo dissociator (Miltenyi Biotec, catalog number: 130-096-427)
9. gentleMACS mixer, octo dissociator (Miltenyi Biotec, catalog number: 130-096-427)
10. Incubator (Panasonic, catalog number: MCO-18ACL-PA)
11. Novocyte 3000 (Acea, catalog number: 2010011)

## Software

1. FlowJo (Version 10.8.0)
2. NovoExpress (Agilent)
3. Prism (GraphPad, Version 9.2.0)

## Procedure

- **Intranasal challenges**
  1. Prepare challenge solution at the correct dosage by diluting*Alternaria alternata*or recombinant IL-33 in sterile PBS.
*Notes:*
    - *Alternaria alternata concentration may vary depending on the lot potency. With new lots of Alternaria, multiple concentrations should be tested to determine the optimal concentration. In our models of*Alternaria*challenge, we typically use 25–50 μg per 40 μL. For models using recombinant IL-33, concentrations of 10–50 ng per 40 μL are recommended.*
    - *The volume prepared should be reflective of challenges to be completed within a one-week period. For example, with a three-day challenge model, the volume prepared would be enough for all three days of challenge.*
  2. Mix the solution well and aliquot for each day of challenge. Additional aliquoted solutions should be stored at -20 °C until needed.
*Note: Before challenging, the solution should be completely thawed and mixed well.*
  3. Anesthetize 6–8-week-old female C57BL/6 wild-type mice one at a time using inhaled isoflurane. Mice are properly anesthetized when their breathing is slow and regular.
*Note: If overly anesthetized, the mouse will begin bucking its head. If this occurs, remove the mouse from the isoflurane and allow it to recover before placing it back in the isoflurane chamber.*
  4. Remove the anesthetized mouse from the isoflurane chamber and pipette 40 μL of challenge solution onto its nose.
*Notes:*
    - *The mouse should be held in an upright position until all of the challenge solution has been inhaled.*
    - *To avoid bubbling of the solution and/or the mouse sneezing out the solution, the challenge should be timed to match the mouse’s breathing (i.e., pipette the solution when the mouse is inhaling).*
  5. Repeat the steps above for all challenge days of the model.
*Note: The timing of the challenges should be consistent each day.*
- **Tissue collection**
  1. Prepare RPMI and 2% BSA in PBS. Keep reagents on ice for the tissue collection.
  2. Euthanize the mouse using carbon dioxide.
  3. Expose the trachea and remove any connective tissue around the trachea. Using an 18 G needle, make a hole at the top of the trachea.
*Note: The needle should be kept parallel to the trachea to avoid puncturing the back of the trachea.*
  4. Place a 21 G catheter through the trachea and flush 0.5 mL of 2% BSA. Allow approximately 10 s before collecting the lavage from this first draw. Collect the bronchoalveolar lavage (BAL) from the first draw in an individual FACS tube and place on ice.
*Notes:*
    - *The 21 G catheter should be created before euthanizing the mouse by threading the 21 G needle through the catheter tubing.*
    - *The supernatant from the first draw of BAL can be used to measure cytokine levels within the airway using ELISA.*
    - *BAL supernatant should be clear when collected. Bloody BAL may be indicative of lung damage occurring during euthanasia.*
  5. Repeat step B4 four more times using 0.6 mL of 2% BSA. The lavage from these draws should be collected together in a separate tube and placed on ice.
*Note: After collecting the supernatant from the first draw for future ELISA analysis, the cells from all five draws can be pooled together and used for flow cytometry staining. Staining these cells using the granulocyte master mix (below) is helpful for measuring the levels of airway eosinophils and monitoring the extent of lung inflammation.*
  6. Expose the chest and pierce the diaphragm. Cut away the diaphragm and cut through the middle of the rib cage.
  7. Pull open the rib cage to expose the lungs. Collect the lungs in 1 mL of RPMI and place on ice.
- **BAL processing**
  1. Centrifuge all of the BAL samples (first draw and pooled consecutive draws) at 500×*g*for 5 min at 4 °C.
  2. Collect the supernatant from the first draw samples and place in an Eppendorf tube.
*Note: The supernatant from the first draw should be stored at -20 °C and can be used for ELISA.*
  3. Aspirate the supernatant from the pooled consecutive draws samples, taking care not to disturb the pellet.
  4. Using 500 μL of FACS buffer (see Recipes), resuspend the pellet from the first draw and combine with the pellet from the pooled consecutive draws.
- **Lung processing**
  1. Place the lungs in a gentleMACS c-tube with 2.5 mL of lung digest buffer, 50 μL of enzyme D, and 7.5 μL of enzyme A.
*Note: The lung digest buffer is a part of the Miltenyi Lung Digest kit and is prepared per the manufacturer’s instructions. Enzymes D and A are also a part of the Miltenyi Lung Digest kit and are prepared per the manufacturer’s instructions.*
  2. Place the c-tubes in the gentleMACS dissociator and run at the lung_01 setting.
*Note: Tissue can get stuck within the blender top. After removing the tubes from the dissociator, make sure that all of the tissue is in the digestion buffer.*
  3. Incubate the tubes at 37 °C for 30 min with end-over-end mixing.
  4. Place the tubes in the gentleMACS dissociator and run at the lung_02 setting.
  5. Filter the solution through a 70 μm filter and into a 50 mL conical tube. Wash the tubes with 2 mL of RPMI to remove any additional cells.
  6. Centrifuge the tubes at 500×*g*for 5 min at 4 °C. Remove the supernatant and resuspend the pellet in 1 mL of RPMI.
- **Cell counts**
  1. To count the cells in the BAL and lung samples, create a 1:100 dilution by diluting 5 μL of the sample in 495 μL of FACS buffer.
  2. Run 100 μL of the diluted sample on the Novocyte at high speed.
  3. Gate the live cells based on forward scatter (FSC) and side scatter (SSC) and use the gated event total to calculate the cell total in the sample, as shown below (Figure 1).
  4. For the BAL, distribute 1 million cells to a separate FACS tube for surface staining.
*Note: If the BAL cell total is less than 1 million cells, then stain the whole sample.*
  5. For the lung, split the sample into parts based on the desired stains.
    - For surface stains, distribute 1 million cells per stain to separate FACS tubes.
    - For nuclear staining, distribute 2 million cells per stain to separate FACS tubes.
    - For cytokine staining, set aside 10 million cells in a separate FACS tube to culture.
*Notes:*
    - *It is important to include controls for the nuclear and cytokine stains for accurate gating of these populations. Fluorescence minus one (FMO) and/or isotype controls may be utilized, although isotype controls may be more accurate for intracellular stains. Controls follow the same staining protocol as the samples; however, the antibody of interest (i.e., Ki-67, Gata-3, IL-5, and IL-13) are either omitted (as in the FMO) or replaced with the antibody isotype (as per the manufacturer’s information). If using isotype controls, it is important to use the same concentration of the isotype as the original antibody for an accurate comparison.*
    - *Samples can be pooled together for the controls. For example, the controls for the nuclear stains were made by pooling together cells from all the samples. Because samples are being pooled together, only a fraction of the sample volume used is needed in order to ensure that the total cells stained do not exceed the number of cells used for the samples.*
    **Figure 1.**
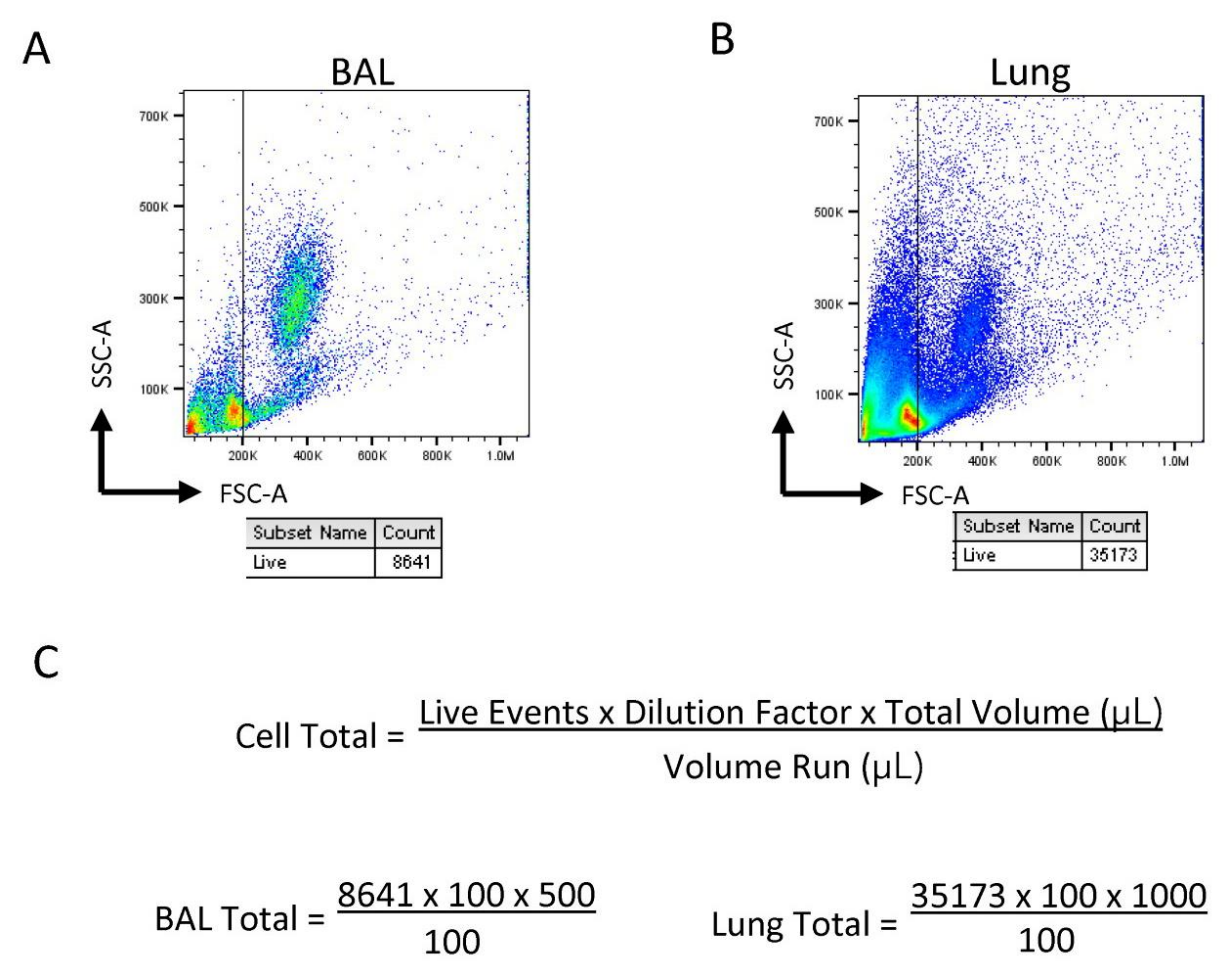
Sample gating and calculation for cell totals. Diluted samples are gated based on size for live cells in (A) bronchoalveolar lavage (BAL) and (B) lung in order to obtain the live event count. (C) Calculation for cell totals within the samples based on event count, dilution factor, and the total volume.
- **Culture for cytokine staining**
  1. Centrifuge the tubes with 10 million cells at 500×*g*for 5 min at 4 °C.
  2. Remove the supernatant and resuspend in 2 mL of T-cell media.
*Note: Important! The T-cell media should be at room temperature when used and should be kept sterile.*
  3. Add 4 μL of cell stimulation cocktail plus protein transport inhibitor (500×) to each tube and mix well by pipetting the solution.
  4. Distribute 200 μL of the solution per well within a flat bottom 96-well plate.
*Note: The goal is to culture 10 wells with 1 million cells per well in cell stimulation cocktail and T-cell media. The volumes noted above are calculated for a starting cell total of 10 million; however, these can be modified depending on the number of cells needed for the cytokine stains.*
  5. Incubate the plate at 37 °C for 3 h.
*Note: During this incubation period, the surface stains and nuclear stains can be completed.*
  6. After 3 h, collect the cells for each sample by pooling the wells together.
  7. Centrifuge at 500×*g*for 5 min at 4 °C, remove the supernatant, and resuspend in 1 mL of RPMI.
  8. Split the sample evenly between the tubes for the cytoplasmic stains and isotype controls.
*Note: These samples are now ready to be surface stained and intracellularly stained as described below.*
- **Surface staining**
  1. Wash the tubes with 400 μL of FACS buffer and centrifuge at 500×*g*for 5 min at 4 °C.
  2. Remove the supernatant and resuspend in 50 μL of FACS buffer with Fc block for approximately 10–15 min.
*Note: During this incubation, the master mixes for the surface stains can be prepared as detailed below.*
  3. Add 50 μL of the master mix consisting of FACS buffer and the appropriate antibodies for each stain, as noted below.
    - Granulocyte: CD11c (FITC), SiglecF (PE), CD45.2 (PerCP), GR-1 (APC)
    - ILC: Lineage (FITC), CD45.2 (PerCP), Thy1.2 (APC)
      Lineage consists of lineage cocktail, CD11c, NK1.1, CD5, FceR1a, TCRβ, and TCRγd
  4. Cover samples in foil and keep at 4 °C for 30 min.
*Note: While staining, compensation beads can be prepared for each fluorochrome used, by staining with 100 μL of FACS buffer, 25 μL of compensation beads, and 1 μL of an antibody in the selected fluorochrome for 15–30 min.*
  5. Wash samples with 400 μL of FACS buffer and centrifuge at 500×*g*for 5 min at 4 °C.
  6. Remove the supernatant and resuspend the samples with the surface stain only in 150 μL of FACS buffer. Surface-stained samples can now be run on the Novocyte.
*Notes:*
    - *Samples that need to be intracellularly stained (nuclear or cytoplasmic) will be permeabilized and stained as noted below.*
    - *Important! If samples will be run the next day, the cells should be fixed in 100 μL of 4% PFA for 15 min. Then, they can be washed and resuspended in 150 μL of FACS buffer.*
- **Nuclear staining**
  1. Prepare the working solution of the FOXP3 permeabilization kit by combining one part concentrate and three parts diluent.
  2. Add 300 μL of the working solution to each sample and place in the dark at room temperature for 30 min.
*Note: Important! Mix the samples well with the working solution.*
  3. Wash the samples with 400 μL of FOXP3 permeability buffer (1×). Centrifuge at 500×*g*for 5 min at 4 °C and remove the supernatant.
  4. Stain the samples with 100 μL of FOXP3 permeability buffer (1×) and the nuclear antibody (i.e., Ki-67, Gata-3, or other transcription factors of interest). Cover samples in foil and keep at 4 °C for 30 min.
  5. Wash the samples with 400 μL of FOXP3 permeability buffer (1×). Centrifuge at 500×*g*for 5 min at 4 °C and remove the supernatant. Resuspend in 150 μL of FACS buffer. Nuclear-stained samples can now be run on the Novocyte.
- **Cytoplasmic staining**
  1. Add 300 μL of the BD Cytofix/Cytoperm working solution to each sample and place in the dark at 4 °C for 20 min.
  2. Wash samples with 400 μL of BD permeability buffer and centrifuge at 500×*g*for 5 min at 4 °C.
  3. Remove the supernatant and resuspend in 100 μL of BD permeability buffer and the cytoplasmic antibody (i.e., IL-5 and IL-13). Cover samples in foil and keep at 4 °C for 30 min.
  4. Wash the samples with 400 μL of permeability buffer. Centrifuge at 500×*g*for 5 min at 4 °C and remove the supernatant. Resuspend in 150 μL of FACS buffer. Cytoplasmic-stained samples can now be run on the Novocyte.

## Data analysis

**Figure 2.**
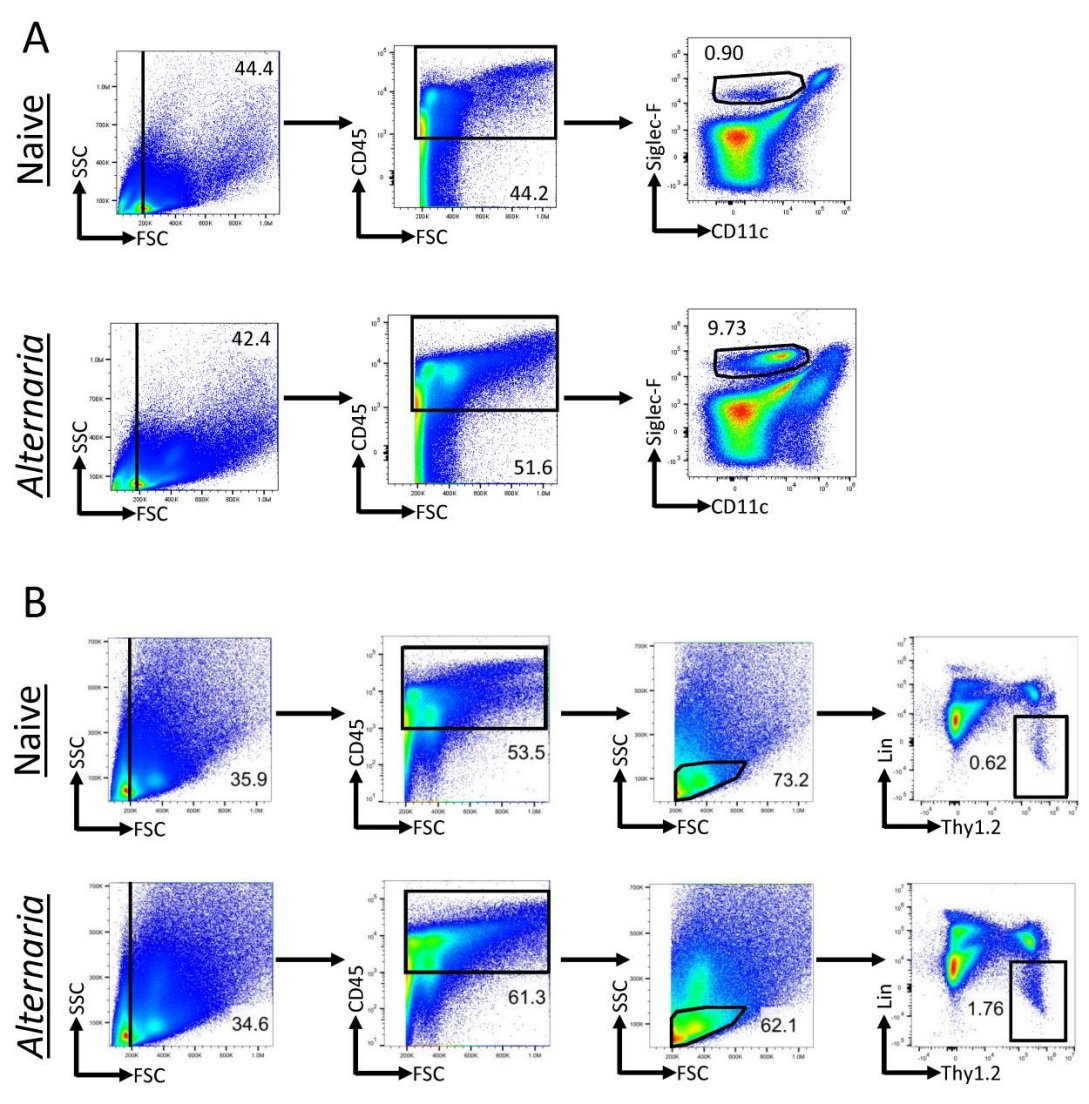
*Alternaria*challenge results in increased eosinophils and innate lymphoid cells (ILCs). *Alternaria*-challenged mice were challenged with 25 μg of*Alternaria*for three days (days 0, 1, and 2) and were sacrificed on day 3. (A) Gating scheme for the identification of eosinophils within lung tissue of naïve and*Alternaria*-challenged mice. Eosinophils were identified as CD11c-SiglecF^+^. (B) ILC gating scheme within naïve and*Alternaria*-challenged mice. ILCs were identified as Lineage-Thy1.2^+^lymphocytes. The lineage cocktail consists of markers for granulocytes, mast cells, B cells, T cells, macrophages, and NK cells, which are all excluded when gating for the lineage-negative population. Data representative of four mice per group.

**Figure 3.**
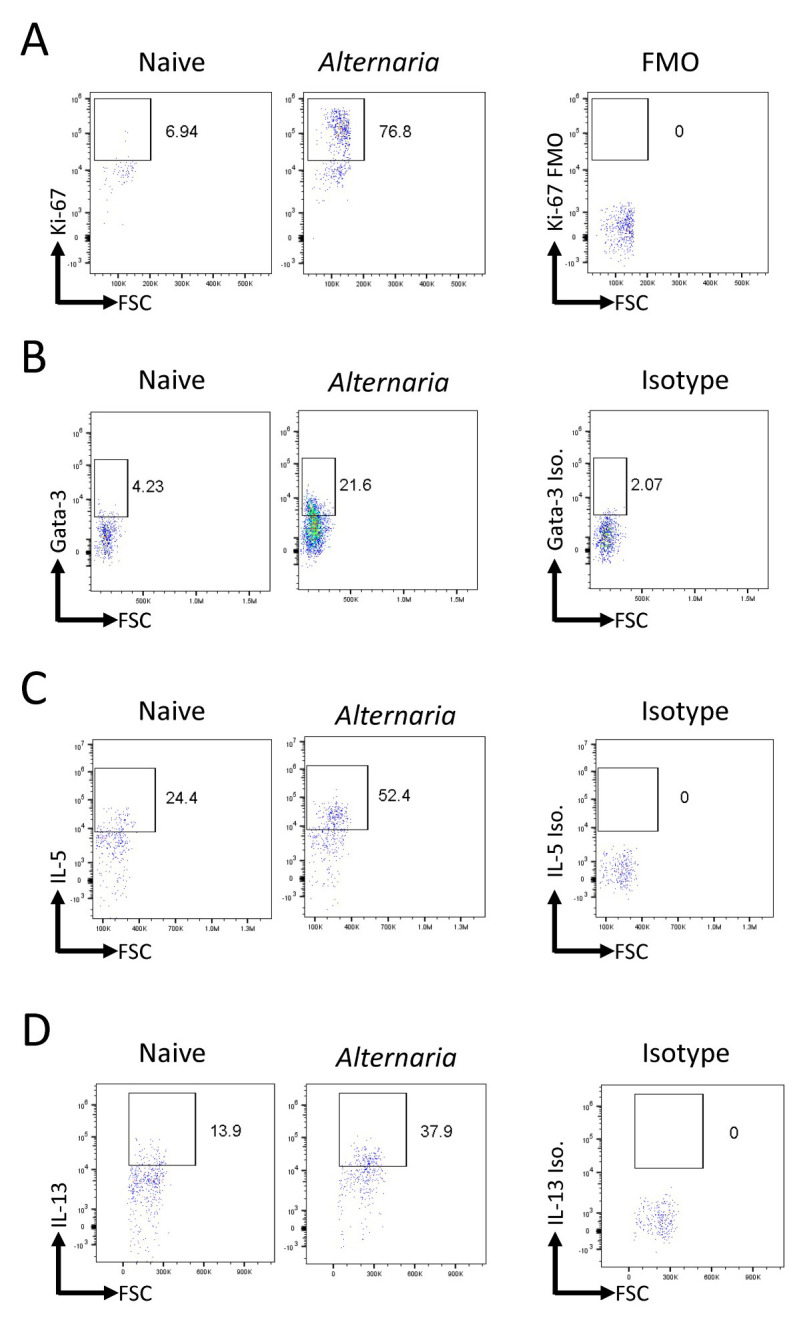
*Alternaria*challenge results in an increased innate lymphoid cells (ILC) population with greater proliferation and Th2 cytokine production. *Alternaria*-challenged mice were challenged with 25 μg of*Alternaria*for three days (days 0, 1, and 2) and were sacrificed on day 3. (A) Representative FACS plots of Ki-67 expression within ILCs in naïve and*Alternaria*-challenged mice compared to FMO control. (B) Representative FACS plots of Gata-3 expression within ILCs in naïve and*Alternaria*-challenged mice compared to isotype control. (C and D) Representative FACS plots of IL-5 and IL-13 levels within ILCs in naïve and*Alternaria*-challenged mice compared to isotype controls. Data representative of four mice per group.

Analysis of FCS files was completed with FlowJo. All populations were gated off of the live CD45^+^population. Live cells were determined based on size (FSC vs. SSC). Eosinophils were identified as CD11c-SiglecF^+^(Figure 2A). Neutrophils were identified as SiglecF-Gr-1^+^. ILCs were identified as Lineage-Thy1.2^+^lymphocytes (Figure 2B). Transcription factor expression and cytokine production within the ILC population was gated relative to an isotype or FMO control (Figure 3).

## Recipes

1. **FACS buffer**
*Note: Stored at 4 °C; use within six months of preparation.*
  1× PBS
  2% FBS
  0.02% sodium azide
2. **T-cell media**
*Note: Stored at 4 °C; use within four weeks of preparation.*
  RPMI
  10% FBS
  1% Penicillin/streptavidin
  1% glutamine
  0.05 mM β-Mercaptoethanol
